# “The Awesomeness and the Vastness of Who You Really Are:” A Culturally Distinct Framework for Understanding the Link Between Spirituality and Health for Black Sexual Minority Men

**DOI:** 10.1007/s10943-021-01297-4

**Published:** 2021-06-17

**Authors:** Jonathan Mathias Lassiter, Ivie Mims

**Affiliations:** 1 Department of Psychology, Rowan University, 201 Mullica Hill Rd, Glassboro, NJ 08028 USA; 2 Department of Psychology, Muhlenberg College, 2400 W. Chew Street, Allentown, PA 18104 USA

**Keywords:** Spirituality, Health, Mechanisms, Black gay and bisexual men, Black psychology

## Abstract

Despite health inequities, many Black sexual minority men are resilient and often utilize spirituality as a culturally distinct self-protective and self-enhancing resource to maintain their health. However, little is known about how spirituality impacts health within a cultural framework that is specific to Black sexual minority men. We conducted 10 individual in-depth interviews, reaching code saturation, with Black sexual minority men across the USA. Our study was guided by grounded theory and a Black psychology theoretical framework. Seven themes were discovered and revealed that participants’ level of spiritual consciousness influenced their engagement in psychological and behavioral processes that were related to mental and physical health. These themes were: (a) suboptimal worldview, (b) emotional revelation, (c) emotional emancipation, (d) emotional regulation, (e) health motivations, (f) health behaviors, and (g) links between spiritual consciousness, mental health, and physical health. Implications of these findings for clinicians and researchers are discussed.

## Introduction

Many Black sexual minority (i.e., gay, bisexual, same-gender-loving, same-sex attracted, and men who have sex with men) men (BSMM) are subjected to heightened psychosocial and political stressors that contribute to them experiencing health inequities. Their intersecting identities (e.g., Black, same-sex attracted) are often marginalized in ways that put them at risk for myriad forms of intersectional discrimination (e.g., racism in White gay communities, homonegativity in Black religious spaces; Balsam et al., 2011; Ward, 2005) and cross-cutting societal barriers (e.g., police and law enforcement interactions, poverty; Brewer et al., 2014; Mena et al., 2016). Individually and in combination, these stressors negatively impact many BSMM’s mental and physical health. For example, psychological distress (Carter et al., 2017; Graham et al., 2011) and HIV/AIDS (Saleh et al., 2016) are but a couple of commonly reported inequitable health outcomes among this group that have been linked with those stressors.

Despite health inequities, many BSMM are resilient. They utilize both mainstream and culturally distinct self-protective and self-enhancing resources. These resources help them to maintain good health and prevent or cope with poor health (Follins & Lassiter, 2017). Spirituality is one such resource that many BSMM utilize toward these aims (Follins et al., 2014). Spirituality is generally defined as a relationship with the sacred, or divine, that transcends the multiple dimensions of the material and ethereal worlds (Lassiter et al., 2020). It is unrestricted by the boundaries and doctrine commonly associated with religion (Miller & Thoresen, 2003); however, it may encompass religious beliefs and practices. The sacred or divine has been conceptualized as being an energetic force that one comes to know in an extrasensory manner (Myers, 1993). It connects one with the life-giving energy in all things (Parham et al., 2016).

Research findings suggest the sacred is an important part of many BSMM’s lives. The sacred is experienced via formal (i.e., religion; Lefevor et al., 2020; Poteat & Lassiter, 2019) or personal formats (i.e., spirituality; Lassiter, 2016; Lassiter et al., 2017). For example, Lassiter and colleagues (2017) found that 80.7% of the BSMM in their sample were either religiously affiliated (55.7%) or identified as spiritual but not religious (25%). Spirituality, the focus of this article, has been found to be related to positive mental health outcomes such as resilience (Lassiter et al., 2019) and negative health outcomes such as depression and substance use (Watkins et al., 2016) among BSMM. For example, among a nationally recruited sample of 1071 sexual minority men (SMM), as spirituality scores increased so did resilience scores and as spirituality scores increased, depression scores decreased (Lassiter et al., 2019). For many BSMM their spirituality is often influenced by their intersecting racial and sexual cultural experiences (Follins & Lassiter, 2017). However, little is known about *how* spirituality impacts health within a cultural framework that is specific to BSMM. This article aims to address this gap.

### Mechanisms That Explain the Associations Between Spirituality and Health

Research with predominately or exclusively White American samples and (presumed) heterosexual Black American samples has contributed greatly to the understanding of the underlying mechanisms that explain the associations between spirituality and health. Some researchers have identified social, behavioral, psychological, and biological mechanisms that mediate the relationship between spirituality and health (Cheadle et al., 2015; Seybold, 2007). For example, empirical evidence suggests that spirituality may impact health by increasing tangible and instrumental support (Chatters et al., 2002) and general levels of social support and integration (Barnes & Hollingsworth, 2020; Le et al., 2016). Spirituality may promote spiritual behaviors (e.g., prayer and spiritual rituals) that induce alterations in one’s internal emotional and physiological state (e.g., equanimity and inner peace; Cheadle et al., 2015). In addition, studies have suggested that higher levels of spirituality are associated with better diet, exercise, and health practices (Ellis et al., 2015). Spirituality may influence health by facilitating meaning-making (Mattis, 2002; Miller, 2019) and use of psychosocial resources (i.e., positive affect and emotional regulation; Mattis et al., 2017) that help people navigate difficult health conditions. There is also evidence that spirituality directly influences biological markers associated with health (Seeman, et al., 2003; Seybold, 2007) such as GABA, melatonin, serotonin (Roberts, 2006), and cortisol (Ironson et al., 2002). Overall, there is a body of research literature that indicates that the mechanisms of action for spirituality and health are numerous and complex.

### Spirituality–Health Mechanisms Research with BSMM

There are two major gaps in the research literature related to spirituality–health mechanisms: (a) a lack of a focus on BSMM and (b) the assumption of a universal spirituality that influences health for BSMM. Few studies have examined spirituality–health mechanisms for BSMM specifically. These studies have tended to focus on sexual health. Foster et al. (2011) found that spirituality helped BSMM living with HIV cultivate psychosocial resources, such as optimism and grit, that contributed to their ability to cope with HIV. Dangerfield et al. (2019) found that, among their sample, spirituality fostered self-love and respect for the sacred nature of sex which, in turn, contributed to participants engaging in positive health behaviors (e.g., using condoms during sex, reducing number of sexual partners). Some participants also endorsed viewing engagement in condomless anal sex as a way to achieve sacred connection with a partner. This lack of condom use could in some, but not all, circumstances constitute health risk behavior. Studies examining spirituality–health mechanisms among BSMM have predominately focused on HIV and sexual risk. There is a dearth of research that examines spirituality–health mechanisms among BSMM beyond sexual health. This article aims to address this gap.

Research about spirituality–health mechanisms, even when inclusive of BSMM, may not be culturally appropriate. Most research in this field has not focused specifically on BSMM’s experiences (Halkitis et al., 2009; Hays & Aranda, 2016). Studies tend to assume that a universal or culturally non-distinct spirituality is influencing BSMM’s health (Carrico et al., 2006). This assumption is not necessarily true. Lassiter and colleagues (2020) found that the BSMM in their sample engaged in a culturally specific spirituality (CSS) that was directly tied to their lived experiences of intersectional oppression and privilege. CSS was identified as having universal, culturally specific, and representational components that combined in distinct ways among some BSMM (Lassiter et al., 2020). BSMM in that study reported:

Understanding their spirituality as (a) a relationship with something greater than themselves, (b) part of themselves, (c) a guiding force in their lives, and (d) multidimensional in nature. The culturally specific components of their spirituality were identified as being (a) an energetic union of masculine and feminine energy within their physical body, (b) connected to their ancestors, (c) an integration of the divine and the sensual, and (d) the connection of spirituality to intersectional oppression (Lassiter et al., 2020, p. 21).

Some BSMM also reported perceiving the sacred as “an energetic, genderless divine form” while other described having multiple versions of the sacred in their minds (Lassiter et al., 2020, p. 22). These components of spirituality were found to be consistent with a Black Psychology theoretical framework that emphasizes the spiritual nature of life.

### A Black Psychology Framework and Its Implications for BSMM’s Health

Black Psychology is a distinct discipline of social science that is understudied but has the potential to help explain how spirituality may influence BSMM’s health. Black Psychology prioritizes precolonial African values such as collectivism, interdependence, and spirituality (Myers, 1993). Theorists and practitioners within the discipline of Black Psychology acknowledge that spirituality is a multidimensional force that constitutes the essence of human beings, with one’s thoughts, emotions, and actions being tools of spiritual development (Akbar, 1996). Succinctly, the Black Psychology theoretical framework posits that life happens within a spiritual context. Spirituality is both a force that a person lives inside and embodies within. Thus, all experiences and actions have a spiritual quality that has implications for one’s health.

Within a Black Psychology theoretical framework, particularly Myers’ Optimal Conceptual Theory (OCT; Myers, 1993), one’s level of spiritual consciousness (i.e., level of attunement to the spiritual nature of life) can fluctuate along a continuum with optimal worldview at one end and suboptimal worldview at the other (Obsai et al., 2009). Myers describes the optimal worldview as prioritizing positive interpersonal relationships, emphasizing the union of opposites (e.g., having a both/and logic), understanding one’s self-worth to be intrinsic, and that all life is interrelated through human and spiritual networks (Myers, 1993). The spiritual component of Myers’ OCT is most relevant for the current article (see Myers et al., 2018 for a detailed overview of OCT). Optimal worldview is theorized as having high attunement to the spiritual nature of life (Myers, 1993). Suboptimal worldview is theorized as a severely low level of awareness of one’s spiritual essence (Myers, 1993). An optimal worldview has been found to be associated with positive health outcomes. For example, the association between stress and depressive symptoms among Black American people was weaker for those with stronger optimal worldview compared to those with a suboptimal worldview (Neblett et al., 2010). Possessing an optimal worldview was also found to be negatively associated with avoidant coping (e.g., denying one’s emotions) and depressive symptoms (Neblett et al., 2010). These findings suggest that an optimal worldview has the potential to not only prevent poor health but also buffer the effect of negative events on health.

In this article, the authors apply OCT to sexual minorities to develop a more holistic and culturally specific understanding of BSMM’s health. OCT would suggest that BSMM’s health is, at least partly, determined by their ability to develop a spiritual consciousness and allow that consciousness to inform their actions, emotions, and thoughts. Given the fluid nature of spiritual consciousness (i.e., it moves along a continuum of optimal and suboptimal worldviews), BSMM’s health may change depending on their fluctuating levels of spiritual consciousness. Thus, maintaining health would rely on retaining an optimal worldview. This Black Psychology theoretical framework has the potential to allow for an understanding of BSMM’s health in a more holistic manner, moving beyond the biopsychosocial to also include the spiritual as a central organizing component.

### Study Aim, Design, and Research Question

This article outlines an investigation of spirituality–health mechanisms among BSMM within a culturally specific framework designed to center the distinct lived experiences and worldview of this group. Qualitative methods, mostly informed by grounded theory methodology (Charmaz, 2014), were selected to guide study design, execution, and analyses as the authors aimed to describe the development of a theory of the spiritual, psychological, and behavioral mechanisms that link CSS with health for a nationally recruited sample of BSMM who participated in the Spirituality Everyday, Everywhere (SEE) Study between March 2017 to November 2017. The primary research question was: What are the culturally distinct mechanisms that link spirituality and health for BSMM?

## Method

### Eligibility Criteria and Recruitment

To be included in the study, participants had to endorse (a) being at least 18 years of age or older, (b) self-identifying as Black or African American, (c) being romantically attracted to men or having had engaged in sexual behavior with a man in their lifetime, (d) identifying as a male, (e) having a current spiritual identity and/or practice(s), (f) currently residing within the USA, (g) having reliable and secure access to the Internet, and (h) possessing the (technological and cognitive) capabilities to engage in video conferencing. Congruent with previous research with SMM (Merchant et al., 2017), a convenience sample was recruited using two methods. The first was face-to-face recruitment at social- and community-based organizations that provided services to BSMM in cities with large populations of BSMM, where the first author had personal contacts. The second method was advertising using banner ads on the Internet and social media platforms that have a large membership of BSMM such as Facebook pages, Instagram, and Twitter posts. In both methods of recruitment, participants were either given digital or paper materials describing the study. The digital materials included a link that led directly to a survey questionnaire on Qualtrics which was used to assess potential participants’ interests in the study. The survey link was printed on the paper recruitment materials.

The survey allowed potential participants to express interest in the study and provide their name and contact information. The first author then followed up with potential participants who had completed the survey. The first author screened individuals by phone for eligibility and provided clarifying information regarding the study. Thirty-five men were screened. Twenty-five men were not enrolled in the study because they were unable to be reached by phone (*n* = 14), had non-working numbers (*n* = 8), reported not being interested after being screened (*n* = 2), and never scheduled an interview time (*n* = 1). The final sample was comprised of 10 BSMM who were deemed eligible and invited to participate in the study. They were assigned an appointment to conduct the qualitative interview. Prior to the men's participation in the interview, they completed a document for informed consent. This project was ethically approved by the institutional review board at Muhlenberg College.

### Qualitative Interview Procedures

Each participant was individually contacted by the first author during the agreed upon appointment time slot. Participants were interviewed using an interview protocol (created by the first author) informed by an extensive literature review pertinent to Black spirituality (Chaney, 2008; Wheeler et al, 2002), Black psychology (Akbar, 2003; Myers, 1993), and spirituality–health research (Smallwood et al., 2015). The findings presented in the current article were obtained from participants’ responses to a subset of five open-ended questions from the 10-question protocol. These questions include the following: (a) How does your spirituality influence your mental health? (b) How does your spirituality influence your physical health? (c) What are the positive and negative aspects of your spiritual life? (d) How has your spirituality failed you? and (e) What are some important things I should know about the way your spirituality influences your health that I did not ask about?

The first author conducted each individual interview using Zoom, a HIPAA-compliant web and video conferencing online platform. The first author video called participants which helped to facilitate rapport during the interview. While Zoom digitally recorded video and audio of the interview, the first author took his own notes (memos) during and between interviews. Memos during the interview were typically short and documented interesting points made by the participant. These memos were used to guide follow-up questions that allowed for more specific elaboration about each primary question (those outlined above) in the interview. Memos completed between interviews documented initial impressions of the participant’s story, the interviewer’s emotional and cognitive responses, the ways in which patterns and concepts from that interview connected with previous interviews, and methodological decisions (e.g., “This participant mentioned […] I should ask the next participant about […] as well). In this way, the first author was able to modify the interview in small ways to focus data collection, engage in reflexivity, and identify emergent patterns in the data. All video data were destroyed after the interview and the audio files were professionally transcribed. On average the interviews lasted for 52 min (range = 32 min to 72 min). Participants received a $10 Amazon gift card as an incentive for completing the interviews.

### Data Analysis

The transcripts were reviewed and compared to original audio recordings to confirm the transcripts’ accuracy and to (re)familiarize the data analysis team (led by the first author) with each interview and participant. Our data analysis was guided by grounded theory methodology (Charmaz, 2014). The data analysis team used line-by-line coding and constant comparison methods to analyze each transcript individually. This process helped us to start to make meaning of each participant’s reality. After initial line-by-line coding of the data, we moved to open (i.e., breaking the data down into distinctive emerging concepts) and axial coding (i.e., building broader concepts from those derived during the open coding phase; Corbin & Strauss, 2014) while still engaging in constant comparisons between and within participant interviews. These methods were employed with the first six transcripts to develop both descriptive and conceptual codes.

During the coding of initial transcripts, the research team noticed significant overlap in codes. By the sixth interview, new codes ceased to emerge (Hennink et al., 2017). These codes were discussed by the research team. Discrepancies during coding were resolved through dialogue until a consensus was reached. The agreed upon codes were formalized in a codebook that was then used for coding the remaining transcripts. We continued to code the remaining transcripts to verify saturation. The reoccurrence of similar concepts and links between concepts that were found in the first author’s memos and the research team’s initial coding, in addition to a lack of new codes emerging suggested that saturation had been reached in the remaining transcripts. The data analysis team met to verify consistency in coding of the remaining transcripts. Similarly, any discrepancies in coding were resolved through discussion until a consensus was reached.

After all codes were identified and agreed upon by all members of the data analysis team, the first author extracted and further coded, using open and axial coding, the sections of transcripts that had been broadly coded: (a) spirituality and mental health, (b) spirituality and physical health, (c) positive aspects of spiritual life, (d) negative aspects of spiritual life, (e) failure of spirituality, and (f) spirituality and other important things. Finally, the first author utilized inductive and deductive strategies (based on intersectionality theory, Black Psychology theories, and spirituality–health mechanisms theories) to develop themes based on the coded transcript extracts. During this process, NVivo 12 qualitative software (QSR International Pty Ltd. Version 12, 2018) was used to store and organize codes into themes.

The data analysis team included three cisgender BSMM. All of them were social science and public health doctoral-level researchers who, combined, have over 40 years of experience conducting qualitative and quantitative research with BSMM. We approached the study with an awareness of the potential biases that sharing similar social identities with our participants might introduce to the process. Such biases included having worldviews that prioritized our spiritual and Black cultural interpretation of our personal and professional lives (see Lassiter et al., 2020 for more details). The first author led the research team and the writing of the current article. The other members of the research team did not contribute to the writing of the present article. An undergraduate Black bisexual cisgender woman who was a research assistant in the first author’s laboratory at the time of this research co-authored the present article. She contributed to writing the literature review, method, and reference sections. She approached the project being personally familiar with and fond of Black American culture. To mitigate this bias in her contribution to this article, she documented when and how her affinity for Black culture may have been influencing her insights and writing. She discussed these with the first author and efforts were made to decrease the effect of her bias.

To assure our biases were not unduly influential to the analyses, results, and interpretation, we reflected upon our own biases in coding meetings, used consensus (described above), and member checks. Member checks (Shenton, 2004) involved emailing a list of extracted themes and supporting quotes (with participants’ individual identifying information removed) to participants. Their attitudes about data appropriateness (i.e., themes matched what participants intended to communicate, themes were rational and descriptive) were solicited and participants responded in writing via email. Seven participants responded with feedback. Participants provided feedback about wording and whether they thought a quote belonged in one category or another. All feedback provided by the participants were incorporated into the analysis. The methods for this study were selected to allow the researchers to acquire a new understanding of concepts rarely studied among BSMM (i.e., spirituality, health, precolonial African values) and to build a model linking these concepts among a very specific group of spiritually conscious BSMM.

## Results

Ten BSMM from seven US states (i.e., Alabama, California, Maryland, New York, Ohio, Rhode Island, and Tennessee) participated in the study. They ranged in age from 26 to 47, with a mean age of 34.3 (SD = 7.3). All men identified as having primarily or solely same-sex romantic and sexual attractions. All men reported engaging in daily spiritual practices (e.g., praying, listening to music that induced a feeling of being connected with the sacred, and reading sacred texts) and that spirituality was “important or very important to them” (see Table 1).

**Table 1:** Sociodemographic characteristics of Black Sexual Minority Male participants

Participants	Age	State of residence
Participant 1	35	Alabama
Participant 2	30	Tennessee
Participant 3	41	California
Participant 4	26	New York
Participant 5	47	California
Participant 6	41	New York
Participant 7	34	Maryland
Participant 8	37	Ohio
Participant 9	26	Rhode Island
Participant 10	26	New York

Note: *N* = 10

**Fig. 1:**
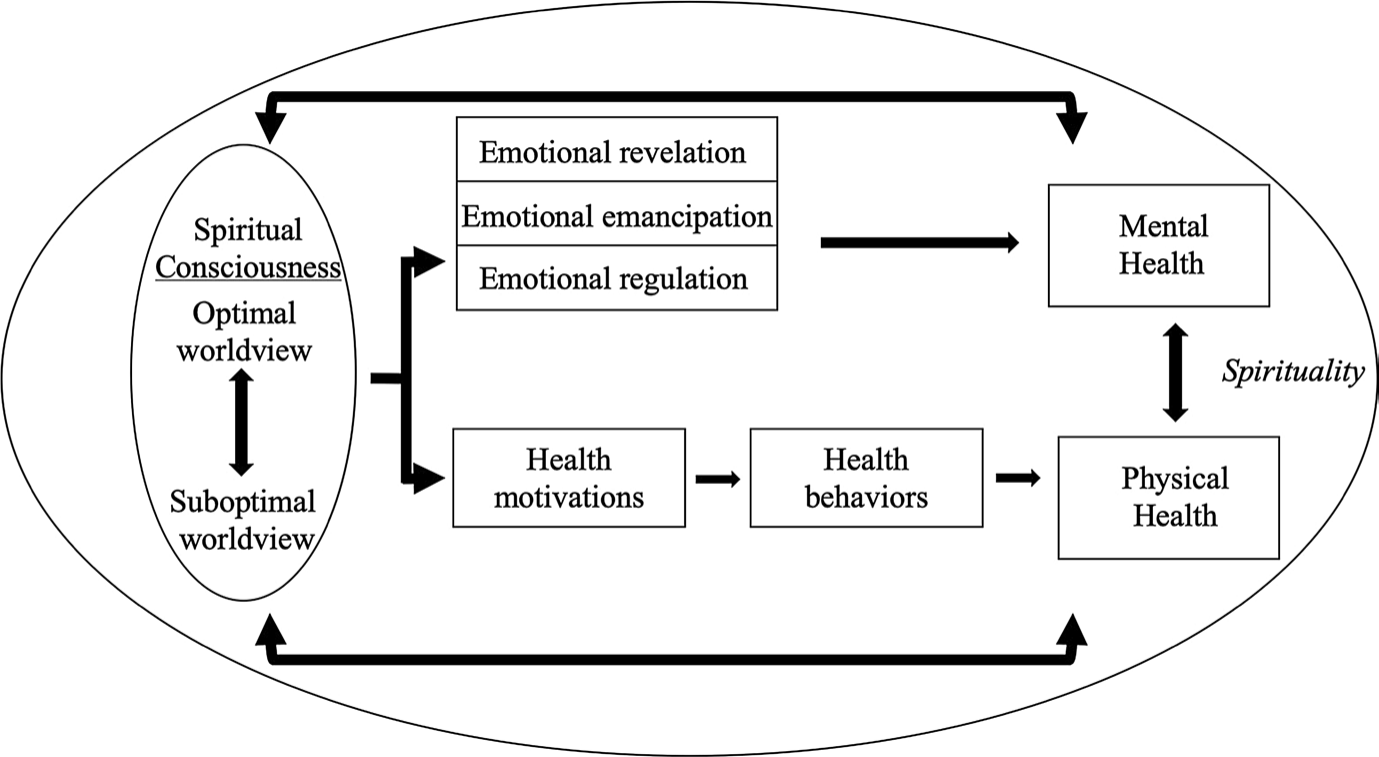
Conceptual model of the pathways through which culturally specific spirituality influences health for Black sexual minority men

We identified seven themes that described the pathways through which spirituality influenced the health of the men in our sample. See Fig. 1. These themes are detailed below.

### Theme 1: Suboptimal Worldview

Eight out of the 10 participants reported possessing a suboptimal worldview at various points throughout their lives. Possessing a suboptimal worldview represented a barrier to BSMM progressing toward good mental and physical health by interfering with their ability to experience their spirituality in a way that promoted health-related psychological and behavioral methods to care for themselves. A suboptimal worldview for BSMM in this sample manifested as a weakened connection to spiritual consciousness due to restrictive religious socialization; negative cognitive, emotional, and behavioral states; and ignoring the sacred. The suboptimal worldview was distinguished by its function of disconnecting, momentarily or long-term, BSMM from their embrace of the spiritual nature of life and their spiritual resources. In those moments of disconnection, participants reported experiencing states of confusion, fear, and preoccupation with non-sacred matters.

Some participants shared that their upbringing in traditional religious settings contributed to difficulty understanding their spiritual lives as adults. Participant 2, a 30-year-old man in Tennessee, stated:

I think the confusion for me comes from maybe [being] a little bit jilted from just my development of religious identity, and maybe confusion around how do those two things [i.e., being religious and same-sex attracted] interact and I guess play a role in my identity moving forward. I think that's where the confusion comes for me is …more so how it plays into my life and what it means to me.

Participant 7, a 34-year-old man in Maryland, expressed that his religious socialization hindered his spiritual development and connection to the sacred. The restrictive religious socialization limited his ability to define himself for himself and instead promoted identity fragmentation and inauthenticity:

I can say in the past…my spirituality didn't allow me to figure myself out. It was always what did God want? What does God want from me? What does God want to see me do? What does God want to see me be? And even when I moved to the more progressive forms of Christianity, it was much of the same. …But then as I began to develop…I began to realize that I needed different frameworks, because I would even hear people who be like “well that’s religion.” But I'm like okay…it required—you had to compartmentalize yourself in a way that I was not comfortable doing. And so it was about being God, God, God over here and all of a sudden it was like, okay, you threw all that away to do something else.

Other participants noted that negative emotional, cognitive, and behavioral states limited their ability to connect to and embody spiritual consciousness. Participant 9, a 26-year-old in Rhode Island, stated that his spirituality does not fail him but that fear sometimes consumes him and leads to failure. He disclosed, “I don't know that my spirituality has failed me. …I was talking about fears, talking about how it's [my fears that] failed me.” Participant 6, a 41-year-old man living in New York, recounted how doubt hindered his ability to perceive the power of the sacred: “I think more or less I may have not trusted [my spirituality] to its capacity to do what it's going to do for me, or I limited how it could help me, so then it looked like it failed me.” He elaborated:

…I only can limit God because I can't imagine beyond this perfect “boo thang,” I can only imagine beyond this job, only imagine beyond knowing this much information, then yeah, spirituality has failed me. But that's because I've limited it to only do something, which means my vision to see it [was] limited…

Participant 4, a 26-years-old who lives in New York, talked about how his engagement in emotionally depleting behaviors sometimes negatively impacted his ability to connect with the sacred. He reported,

I think in that sense there have been times where I have given too much…of my time, too much of my energy, too much of my resources to things and to people…I felt like it didn’t yield, like, the return… If anything it took away from me or was more taxing.

Participant 5, a 47-year-old who lives in California, admitted that he sometimes failed to acknowledge the sacred which contributed to his disconnection from it. This disconnecting, in turn, contributed to negative life experiences. He shared, “The only time I think [my spirituality] really has failed me is when I don't use it. When I don't utilize it at all. When I go out on my own and not really bring it in.”

### Theme 2: Emotional Revelation

Five out of 10 participants shared stories that illustrated this theme. Their stories contribute to an understanding of emotional revelation as the awareness of emotions that are difficult to recognize, sit with, or own that is facilitated by one’s spiritual consciousness. Participant 6, a 41-year-old in New York, stated:

…it becomes like these moments of just super clarity like, “Dude, you're depressed. What the hell is that?” Or I'm somewhere, and I'll glance over and I'll see someone and, for me, the divine has allowed them to mirror how I feel on the inside, and then it becomes like, “Oh, that's you. Ugh. You need to fix that.” Or I may be at work and just be in my zone and someone will be like, “Are you okay? Your smile isn't like how we're used to seeing it,” or, “Oh my god, you are really glowing.” Because sometimes, I may not see how I feel, I may not be aware of how I feel at that moment because I'm in automatic [mode]. The divine will send messages to me or I will sense something, because I may watch something so it may cross my eyes, and it may engender the emotion that I've not really understood what was going on inside because I'm busy doing life.

His comments highlight how one’s spiritual consciousness facilitates being able to recognize and identify emotions that one did not originally have the knowledge or language to name. Moments of clarity, other people, and divine messages were reported as tools of the sacred that facilitated emotional revelations among BSMM. Along these lines, Participant 7, a 34-years-old in Maryland, disclosed “Well, now it helps to realize I've been really hard on myself.” Participant 10, a 26-year-old in New York, shared, “if it were not for my spirituality, I would not know who I am. I would not be able to say that I identify as something. I would not know what I believed was the correct way to represent myself and to feel comfortable and to feel safe with myself.” In many ways, emotional revelation was connected to how participants recognized, understood, and reacted to their internal processes and sense of self.

### Theme 3: Emotional Emancipation

Six participants provided data that epitomized this theme. Their stories depicted emotional emancipation as the process through which spiritual consciousness allowed them to fully express or embody their emotions without judgment. Participant 6, a 41-year-old living in New York, stated:

My spirituality has allowed me to embrace my feelings, the whole spectrum, from the most wrathful, vengeful thing, to the most docile and sweet and most loving thing, and be okay with the entire spectrum that neither, technically, is right or wrong but is bringing me back to my center.

Participant 7, a 34-year-old in Maryland, shared, “It allows me to be honest with myself. It's saying, listen, here's what it is.” Participant 10, a 26-year-old in New York, stated:

I don't have the worries of when I go outside. I don't have to feel like I have to work extra hard. I feel that I can be extremely vulnerable. I can be crying, I can be upset, I can even be angry because this is a relationship that I built, so this is something or someone that I can express myself fully to. It's just a life with no barriers and no limits. It just feels complete.

Participant 5, a 47-year-old man in California, disclosed that his spiritual consciousness aided him so that he could accept his emotional experiences and not label them based on stereotypes about his identity as a Black man.

…for me to not beat myself up if I'm in a negative state of mind or beat myself up because I felt the need to express something that some people did not like and not buying into the whole thing of, “Okay, you're just doing that only because you're an angry Black man.” No. You're doing that because that's where you're coming from. That's your experience.

### Theme 4: Emotional Regulation

Nine out of 10 participants disclosed that their spirituality helped them regulate their emotions. Within the context of this analysis, emotional regulation can be understood as the process whereby spiritual consciousness triggers one’s use of emotional, behavioral, cognitive, or social strategies to induce positive emotional states or reduce or prevent negative emotional states. Participant 8, a 37-year-old man in Ohio, revealed that his spiritual consciousness guided his behavior. He said:

By just praying to God and being a better person, and just doing what God would want me to do. Having knowledge of what God would want you to do will lead you to a lot of things. You can actually hear God saying don’t do something. Even in times before when I might have just went off and socked somebody, or something like that, and not thought about it, just did. I don’t be on that no more.

Participant 1, a 35-year-old in Alabama, explained that his spiritual consciousness sometimes triggered cathartic emotional responses or behaviors that helped him cope with distressful emotions. He shared:

When [god] comes in or when I acknowledge that he's there, when I feel his presence, it's like, "Okay, I feel better." Like…after [or] during that time, I may cry. I may scream. I may throw something. I may just go for a walk. But just that moment when I know that he's there, I'm overcome with peace.

Participant 6, a 41-year-old man in New York, reported that his spirituality incites him to decenter himself and focus on his spiritual nature as a way to navigate painful negative emotions. He articulated:

Make yourself small enough so that you can realize the awesomeness and the vastness of who you really are. It kind of brings me out of the funk of kind of being depressed or not beating up on myself because someone done made a comment, and I'm human. I may suck it up for a second, but then when it gets to that place, the divine is like, “Okay, let's go to the park. You know what? Go get on your bike. Go ride. You know what? Just go walk around the complex and put on some music, and then whatever happens, let it happen.”

Participant 3, a 41-year-old man in California, similarly shared that his spirituality facilitated engagement in a cognitive meaning-making process in the face of stressors. He stated:

…this gets into this system of faith and belief…that soul development. That energy development. I look at a lot of things that are going on, or situations that come into my life, and ask what is my lesson in this?

### Theme 5: Health Motivations Informed by Culturally Specific Spirituality

Health motivations denoted cognitive reasoning for health promoting behaviors that were informed by the men’s culturally specific spirituality. Six participants reported that they perceived their physical bodies as physical extensions of the sacred and thus they felt a responsibility to care for their physical health. Participant 7, a 34-year-old man in Maryland, stated:

And I think that's the piece that I'm realizing in my spirituality. What you can do though is put yourself in the best possible position to allow something to happen. And that's what my physical workouts are for me. It's also about recognizing, hey, I'm diabetic now, so there's something added to it. And so I would like to mitigate the impact as much as possible.

Participant 1, a 35-year-old man in Alabama, said:

…because of me being rooted and having a relationship with God and acknowledging who he is, there's a passage of scripture in the Bible that states, "I wish above all things that thou mayest prosper and be in good health, even as the soul prospers." So, I look at that as even as while I'm getting spiritually fed through Sunday services, through Wednesday night Bible class, through Sunday school, or even just listening to music myself, I still have to do what I have to do to keep my natural body healthy. Eat right, proper nutrition, water, things of that nature. So, that's how it influences. There's no point in me talking about some, "I love God, and he's the best friend that I ever had," and I'm not eating and I'm around here fainting and passing out. That's not going to hold.

Participant 8, a 37-year-old in Ohio, also described this theme when he revealed:

The body is God’s temple. By me loving God and loving myself, you don’t wanna tear down your temple. I don’t wanna tear down this temple that God gave me, it’s the only one that I have for right now and I wanna make sure that it’s working. He blessed me with it, I’m gonna make sure that I take good care of it.

### Theme 6: Health Behaviors Influenced by Culturally Specific Spirituality

Two participants expressed that they did not see a connection between their spirituality and physical health. However, four participants explicitly mentioned that their connection to the sacred contributed to them engaging in some form of healthy behaviors or self-care. The health behavior theme highlights the actual concrete health promoting behaviors in which the participants engaged. For example, when asked how an outsider watching him, but not talking to him, would be able to see the presence of the sacred in his life, Participant 8, a 37-year-old living in Ohio, listed the healthy behaviors in which he engaged: “You would see me doing these puzzles—these little brain teaser puzzles that I do all the time to make sure my brain stays sharp. You’ll see me—catch me on my little diet.” Participant 4, a 26-year-old who lives in New York, spoke of how his dancing was a link to the sacred and keeps him healthy.

I vogue, I dance and I think vogue for me has become a form of, like, praise, right? And you know I gotta get my praise on, like, I have to vogue at least once-a-day even if it’s for, like, a minute and even if it’s not, like, a whole vogue, like, if it’s just me, like, in the mirror, playing with my hands or, like, doing the catwalk or something, like, for a few seconds, it’s something, right? It makes me feel better. It feels, it’s cathartic, right? I feel like something about it is very, like, tribal, right?

Participant 7, a 34-year-old in Maryland, discussed how his spirituality helped him persevere in his physical health practices. He reported:

…you commit yourself for five weeks to practicing making timely practice, stretching, running, even when it was a little cold outside, even when it was a little dark out. You really made an effort to prepare yourself, to put yourself in the best spot to do something. And to me that was a spiritual practice. And that to me that leads more to everything else and say, hey, it's not so much—‘cause I can't make anyone—you can’t make anyone do anything for you. And I think that's the piece that I'm realizing in my spirituality.

### Theme 7: Links Between Spiritual Consciousness, Mental Health, and Physical Health

Four participants highlighted the reciprocal links between their mental health, physical health, and spiritual consciousness. Participant 9, a 26-year-old man in Rhode Island, stated:

So understanding that my mental health, like having good mental health is sort of also good for my spirituality, 'cause then I'm able to be more whole and more in a better state of Zen. [Laughs] Then makes me more likely or helps me make better choices that affect my physical health.

Participant 7, a 34-year-old man in Maryland, also expressed that the physical, mental, and spiritual aspects of his health are connected. He shared:

It helps me to—I like feeling physically strong and physically capable. That's a large part of my—that helps me to feel confident. The forging and the focus and the effort in the gym or on the court helps me to feel alive outside of it. But I think the spirituality also about being compassion is like saying, okay, you're 34, not 25. And that means that you have to look at yourself differently and handle yourself differently.

Participant 10, a 26-year-old in New York, also disclosed:

I have just been fasting and fasting and fasting because I know that is gonna get me in a good place emotionally and physically… It keeps me balanced, where I remember to drink water and I remember to eat fruits and not eat Lays and pizza and all these things that taste good but don't make me feel good.

## Discussion

### Summary of Findings

This study utilized qualitative methodology—informed by grounded theory data analytic methods within a Black Psychology theoretical framework—to explore the pathways that link culturally specific spirituality and BSMM’s health. In this conceptual model, spirituality is the context within which all life happens. One’s level of attunement to this spiritual context (i.e., spiritual consciousness) can be strong (i.e., optimal worldview) or weak (i.e., suboptimal worldview). We found that when spiritual consciousness was suboptimal (i.e., representing a suboptimal worldview), participants reported that they failed to acknowledge the sacred. This lack of acknowledgment hindered them from effectively engaging healthy psychological and behavioral processes to positively affect their health. Participants identified restrictive religious upbringings, negative psychological states, and ignoring the sacred as contributing to them possessing a suboptimal worldview.

Participants shared stories that illustrated the mechanisms through which an optimal worldview contributed to their health. Psychological mechanisms included emotional revelation, emotional emancipation, and emotional regulation. Participants described experiences of emotional revelation where possessing an optimal worldview facilitated their abilities to recognize and give language to emotions that had been previously unobtainable or too painful to fully acknowledge. These findings suggest that BSMM’s high attunement to their spirituality may be associated with emotional intelligence (i.e., ability to attend to, interpret, and understand emotional stimuli; Huynh et al., 2018), either contributing to it or being a potential outcome. This finding goes beyond previous research that found associations between spirituality, increased positive emotions (Mattis et al., 2017), and psychological resources (Miller, 2019). Instead BSMM’s spirituality may play a role in not just engendering emotions but also in the process of accessing and comprehending one’s emotions.

Possessing an optimal worldview influenced participants’ mental health by facilitating emotional emancipation (i.e., accepting one’s emotions without judgment). Emotional emancipation seems to be different from the cultivation of a nonjudgmental approach to one’s emotions as facilitated through mindfulness. Mindfulness emphasizes paying attention to the current moment without judgment (Watson-Singleton et al., 2019). Emotional emancipation is not only about the current moment but about all moments converging and, through the power of one’s spirituality, facilitating an acceptance of one’s emotions (past, present, and future). This sort of emotional emancipation may be especially important for BSMM whose emotional expression is often policed by others (Jackson, 2018; Totten, 2015). This emotional policing is sometimes enacted via communication of explicit and implicit gendered racist attitudes (e.g., “Black men are dangerous”), homonegative beliefs (e.g., “Black gay men are ruining the Black community”), and heteronormative prescriptions (e.g., “You’ve got to be tough to survive in the world as a Black man”). Emotional emancipation may allow BSMM to extend themselves beyond the emotional prisons erected by intersectional oppression that stigmatize their identities. Emotional emancipation has the potential to help BSMM view themselves as “fully human, requiring no explanation or justification” (Grills et al., 2016, p. 338). Our findings suggest that an optimal worldview may impact mental health through emotional regulation. This finding is consistent with previous research (Cheadle et al., 2015). Participants’ emotional regulation was enacted through spiritually informed cognitive re/appraisal, emotional coping, behavior change, and social support utilization.

BSMM varied in their interpretations of how spirituality influenced their physical health. Some men recognized a connection, others did not. Among those who did, they recounted stories that described their optimal worldview as influencing their health motivations and behaviors. Most participants reported understanding their bodies to be an extension of the sacred. This conceptualization contributed to them engaging in health behaviors to achieve or maintain good physical health. Our findings are consistent with previous research that highlighted Black Americans’ understanding of their bodies as sacred and in need of maintenance (Lewis et al., 2007). The reciprocal link between spiritual consciousness, mental health, and physical health was also highlighted among participants. All three components worked together to contribute to overall holistic health.

### Clinical and Research Implications

Altogether, our findings lend support for the application of OCT to sexual minorities’ health. Consistent with OCT, our findings indicate that both intrinsic (e.g., negative psychological states, ignoring the sacred) and extrinsic (e.g., restrictive religious upbringings) factors influence one’s spiritual consciousness which, in turn, influences one’s health. This Black psychology-informed framework may facilitate an understanding of BSMM’s health in a more culturally specific manner that highlights the totality and integration of their multiple dimensions (e.g., biopsychosocial-spiritual). Our findings may prove beneficial in helping researchers and healthcare providers working with BSMM conceptualize and address their health concerns in more nuanced and holistic ways.

Our findings underscore the importance of healthcare providers prioritizing developing their knowledge, awareness, and skills (Iverson, 2012) related to addressing spirituality and its connection to BSMM's health. Healthcare providers may benefit from training about BSMM’s spirituality and how these men understand its influence on their health. Healthcare providers should also engage in reflexive activities to interrogate their own beliefs about spirituality, health, and BSMM’s culture. Furthermore, learning culturally relevant skills such as how to conduct spiritual assessments (e.g., FICA [Faith and Belief, Importance, Community, Address in Care] tool; Puchalski, 2013; spiritual ecomaps; Hodge & Williams, 2002) may be helpful in exploring BSMM’s spiritual beliefs and journeys and how they may be incorporated into treatment planning. Healthcare providers may be better able to serve their BSMM clients by familiarizing themselves with therapeutic models and approaches, such as the GRACE therapeutic model (Goals, Renewal, Action, Connection, and Empowerment; Bozard & Sanders, 2017) and Belief Systems Analysis (Meyers et al., 1996), that center the intersections of spirituality, culture, and health. These models and approaches may enhance treatment with spiritually conscious BSMM. These approaches allow for the integration of spirituality into the healing process for BSMM whose poor health may be linked to racial discrimination, religious abuse (Bozard & Sanders, 2017), spiritual genocide and alienation (Schiele, 2000; Ward, 2005), and other forms of harm due to systemic and interpersonal bias.

Our findings are ripe for further exploration and refinement. Future research aimed at increasing the understanding of BSMM’s spirituality–health mechanisms within a Black Psychology framework may benefit from exploring different types of qualitative data and incorporating quantitative data. For example, it may prove beneficial for researchers to add questions that query how BSMM’s spirituality influences specific types of health outcomes (i.e., anxiety, hypertension). Along those lines, it may also be useful to interview BSMM who already have specific health diagnoses (i.e., generalized anxiety disorder, congestive heart failure). These approaches may facilitate participants’ thinking in more concrete and proximal terms about their spirituality and health. In addition, large-scale survey research may allow scientists to test or refine the model proposed in this article with a larger sample.

### Limitations

Although conducted to prioritize theoretical and methodological rigor, this study has some limitations that should be considered. First, readers should avoid generalization of this article’s findings as universal knowledge or representative of a larger sample, as qualitative methods are not designed for that purpose. However, the authors have attempted to obtain analytical generalizability with our efforts to integrate our findings within a Black Psychology theoretical framework to highlight the potential relevancy of OCT for sexual minorities’ health (Hays & McKibben, 2021). We acknowledge that our sample size may be small. However, we did obtain code saturation (i.e., where no new knowledge emerged from subsequent interviews). This is consistent with other researchers’ findings that code saturation can be reached with small samples with 95% of the most salient ideas emerging by the 10th interview (Hennink et al., 2017; Weller, et al., 2018). We conceptualize our findings as preliminary and possibly only applicable to men of a very specific background (i.e., BSMM who acknowledge the importance of spirituality in their lives and who endorse having a daily spiritual practice). This specificity was intentional in the research design as we wanted to understand the mechanisms that linked spirituality and health in ideal conditions of spiritual consciousness. Finally, to reduce participant burden and increase confidentiality with a vulnerable population, we collected limited sociodemographic data. Additional sociodemographic data such as religious denominational affiliation and income may have provided more context for our findings.

## Conclusion

This study breaks new ground despite its limitations. BSMM have culturally distinct articulations of psychological and behavioral mechanisms that link their spirituality and health. Spirituality is an organizing force in which their lives unfold, and their attunement (i.e., spiritual consciousness) to this spirituality (whether optimal or suboptimal) influences the quality of their health. Healthcare providers and researchers who work with this population are encouraged to further explore the culturally distinct aspects of spirituality and health for BSMM, as well as develop skills and programming that center these spirituality–health processes. Health happens in the context of culture. The more culturally attuned health practices and science are, the more effective they will be.

## Data Availability

Data are not available for public use to protect participants’ confidentiality.

## Code availability

A copy of the codebook for this study may be obtained by contacting the first author.
